# Digitally Deprived Children in Europe

**DOI:** 10.1007/s12187-022-10006-w

**Published:** 2023-01-04

**Authors:** Sara Ayllón, Halla Holmarsdottir, Samuel Lado

**Affiliations:** 1 Department of Economics, EQUALITAS & IZA, University of Girona, Girona, Spain; 2 Department of Primary and Secondary Teacher Education, Oslo Metropolitan University, Oslo, Norway; 3 Department of Economics, University of Girona, C/Universitat de Girona 10, 17003 Girona, Spain

**Keywords:** Digital deprivation, Computer, Internet, Poverty, Education, Europe

## Abstract

The COVID-19 pandemic has completely changed the need for internet connectivity and technological devices across the population, but especially among school-aged children. For a large proportion of pupils, access to a connected computer nowadays makes the difference between being able to keep up with their educational development and falling badly behind. This paper provides a detailed account of the digitally deprived children in Europe, according to the latest available wave of the European Union – Statistics on Income and Living Conditions (EU-SILC). We find that 5.4% of school-aged children in Europe are digitally deprived and that differences are large across countries. Children that cohabit with low-educated parents, in poverty or in severe material deprivation are those most affected.

## Introduction

The COVID-19 pandemic has completely changed the need for internet connectivity and for technological devices across the population, but particularly among children. In an attempt to halt the spread of the virus, many countries moved part or all of their teaching online,^1^ accelerating the process of adoption of digital technologies in education, such as blending digital tools with traditional teaching methods (Guallar Artal et al., 2021). Therefore, nowadays, for many children, having a computer connected to the internet makes the difference between being able to keep up with their education and falling badly behind. Issues of access are linked to the larger body of research surrounding digital exclusion, digital inequalities and the digital divide (Chen, 2013; DiMaggio & Hargittai, 2001; DiMaggio et al., 2004; Helsper, 2021; van Deursen & van Dijk, 2010; van Dijk, 2005). Other aspects during the COVID-19 pandemic have also been relevant — such as having the opportunity to stay socially connected with family, friends and peers at a time when physical distancing was imposed in most countries (Ellis et al., 2020; Ezpeleta et al., 2020).

But not all children in Europe have either a computer or an internet connection. As a matter of fact, our results based on data from the European Union – Statistics on Income and Living Conditions (EU-SILC), show that, on average, 5.4% of children in Europe are digitally deprived: that is, they live in a household that cannot afford to have a computer and/or live with adults who claim they cannot afford to have an internet connection for personal use at home. However, the differences across European countries are large. For example, in Iceland, only about 0.4% of children are digitally deprived, whereas in Romania and Bulgaria the figure soars to 23.1% and 20.8%, respectively.

Much of the work on digital inequality — or, more specifically, the digital divide — has focused on access (the ‘first-level’ digital divide), which was assumed to be largely resolved (Paus-Hasebrink et al., 2019; van Deursen & Helsper, 2015). According to van Deursen et al. (2011), ‘the binary classification of access in terms of physical access (having a computer and an internet connection or not) is considered to have been superseded and replaced by a divide that is supposed to concentrate on a large number of more complex variables and relations’ (p. 126). This prompted a move to focus research on digital use and digital competencies, understood as ‘digital skills’ and often referred to as the ‘second-level’ digital divide (Hargittai, 2002; Ronchi & Robinson, 2019). The shift from access to skills and usage was seen as necessary in order to reflect changes in society, where digital skills were becoming more important (van Dijk, 2017). However, the pandemic has shown us that the assumption that ‘now everybody has access to and can use the internet’ (van Deursen et al., 2011, p. 126) is inaccurate; instead, it has served to demonstrate that children still face inequalities in access, leading to digital exclusion — or what we call ‘digital deprivation’.

This paper answers the following questions: Who are the digitally deprived children of Europe? Where do they live? Is the risk of digital deprivation among children heterogeneous across Europe? What are the associated risk factors of children’s digital deprivation? We consider six vulnerable groups: (i) those who live in a lone-parent household; (ii) those who live in a poor family; (iii) those living in severe material deprivation; (iv) those with parents of non-European immigrant origin; (v) those with low-educated parents; and (vi) those in a large family. To the best of our knowledge, no previous study has determined the level of digital deprivation among children in Europe, and nor has any identified the socio-economic characteristics that increase the likelihood of a child being digitally deprived. Understanding who the digitally deprived children are and what the existing differences in risk are across Europe is crucial if we are to design effective policies to combat the digital divide, and if we are to ensure equal educational opportunities for all children in Europe, irrespective of their socio-economic background.

We find that digital deprivation affects particularly children in severe material deprivation, those that cohabit with low-educated parents and those who live in poverty. However, the characteristics that describe a digitally deprived child are heterogeneous across countries (as is the strength of the association). For instance, while cohabiting with parents of non-European immigrant origin is positively associated with digital deprivation in most contexts, this is not the case in Eastern Europe or the Baltic countries, where this characteristic is negatively associated with the probability of being a digitally deprived child. Also, living in a large family is positively associated with digital deprivation in most contexts, apart from in the English-speaking countries.

The section that follows this introduction reviews the existing literature on digital exclusion. Section 3 introduces the data used and our definition of digital deprivation. Section 4 shows our results in terms of the big differences in the prevalence of digital deprivation across European countries. We also provide a detailed account of the individual and household socio-economic characteristics associated with children’s digital deprivation. Finally, Section 5 summarizes the main findings and proposes some policy recommendations.

## Literature Review

The development and increasing use of digital technologies has affected the lives of children and young people, and has, in turn, raised concerns about the emergence of new digital inequalities and the intensification of existing ones. These concerns have led to considerable work that has focused on digital exclusion or digital inequalities — often perceived in terms of a digital divide. The first report on the digital divide from the National Telecommunications and Information Administration (1999) focused on the ‘have nots’ in rural and urban America. It served as a foundation for the initial work on the digital divide. That work was rather technical, pursuing a binary understanding of access, with a focus on demographics (van Deursen et al., 2011). According to Robinson et al. (2020), this techno-deterministic approach to access was seen as oversimplistic, assuming as it did that access alone led to a reduction in inequalities (Katz & Aspden, 1997). This prompted further work on the digital divide and led to a new understanding of the concept, to different definitions and to a focus on different levels of the digital divide (DiMaggio & Hargittai, 2001; Gunkel, 2003; Hilbert, 2011; Selwyn, 2004; van Dijk, 2005, 2006). Despite the heterogeneity of the definitions and levels of the digital divide, for the purposes of this article we adhere to the definition provided by the Organisation for Economic Co-operation and Development (OECD, 2001a), which interprets the digital divide as ‘the gap between individuals, households, businesses and geographic areas at different socio-economic levels with regard both to their opportunities to access information and communication technologies (ICTs) and to their use of the internet for a wide variety of activities’.^2^

Several frameworks exist that seek to explain the dimensions and factors that influence the digital divide (DiMaggio & Hargittai, 2001; Helsper, 2021; van Dijk, 2006). What many of these frameworks have in common is that, in seeking to explain the digital divide, they incorporate one or more of a number of dimensions — material, motivational, skills and usage. Given our attention to digital deprivation, this article focuses on the material dimension. It is concerned with the root causes of deprivation, which are economic in nature (Pacione, 2009). While material deprivation is linked to the complex poverty problem (Pacione, 2009), it is defined as ‘the enforced lack of a combination of items depicting material living conditions, such as housing conditions, possession of durables, and the capacity to afford basic needs’ (Shamrova & Lampe, 2020, p. 2). The material dimension is closely linked to the idea of digital inclusion, which is broadly defined as different strategies designed to ensure that all people have equal access, opportunities and skills to benefit from digital technologies and systems (ITU, 2022). The material dimension, as we see it, is linked to digital deprivation, which is a socio-economic phenomenon that describes the gap in access, but that can also affect usage (OECD, 2000, 2001b). The broader thinking around digital inclusion and the digital divide has evolved over the years and is currently grouped into three categories: binary internet access (first-order digital divide), digital skills (second-order digital divide) and the outcomes of internet use (third-order digital divide) (Scheerder et al., 2017). It is the first-level divide that we are interested in, as it has implications for the other two levels. The outbreak of the COVID-19 pandemic meant that digital exclusion due to lack of access has become more pronounced than ever before. Furthermore, with the growing importance of digital technology in our everyday lives, access to digital technology has become a basic need; and without that access, the gap between the economically rich and poor will most likely increase over time (Helsper, 2021). Nevertheless, ‘for access to be a true indicator of inclusion in digital societies, it should be high quality as well as ubiquitous’ (Helsper, 2021, p. 52). Moreover, of course, without access no one can use the internet (or other digital technology), and so the ability to develop skills, motivation, and general use is impeded.

As van Dijk (2006) points out, the digital divide did start to attract growing attention; but from about 2005, interest began to wane, as the developed countries ensured that a large part of their population had access to electronic devices. However, the COVID-19 pandemic has shown that access issues are still important, and this has reignited the debate among scholars, who again focus attention on this aspect of the digital divide. The recent work includes research by Seifer (2020), Gibson et al. (2020) and Martins van Jaarsveld (2020), all of whom study the effects of the digital divide in the COVID-19 crisis among the elderly population. Among school-aged children, Rodicio-García et al. (2020) find that 14.8% of students in Spain recognize that they do not have enough resources to follow online education, while Stelitano et al. (2020) explain that students of colour who live in great poverty or in rural areas of the US report having had less access to the internet at home during school closures. Furthermore, research by Kuc-Czarnecka (2020) indicates that there are areas of Poland that are especially vulnerable to digital deprivation. It is still hard to predict how the post-COVID-19 world will look (Kufel, 2020), but the increasing pervasiveness of digital technology is a reality that all countries face (Kuc-Czarnecka, 2020).

Aside from the more recent academic research driven by the pandemic, earlier studies analysed several dimensions of the digital divide. For instance, the work of Longley and Singleton (2009) matched the 2004 Index of Multiple Deprivation (IMD) with a classification of ICT usage in the United Kingdom. They suggested that the lack of digital engagement was linked to high levels of material deprivation. In this respect, the authors developed a cross-classification of material deprivation and ICT usage. Yelland and Neal (2013) went beyond the classical digital-divide dichotomy between the ‘haves’ and the ‘have nots’ and looked at how the lives of families in low socio-economic areas of Australia improved as a result of their being given a computer and internet access. Students noted that they could complete school work and communicate with friends, while parents saw an increase in all family members’ confidence and active participation in their communities.^3^ Gordo (2003) found similar results and emphasized that closing the digital gap could benefit people who live in poverty.

Recent research by the International Computer and Information Literacy Study (ICILS) shows that, on average, students with a better socio-economic background have significantly higher Computer and Information Literacy (CIL) scores (Fraillon et al., 2020). Students’ CIL scores are shown to be associated with access to computers at home and years of experience using computers, according to the authors. In all participating countries, students with two or more computers at home have statistically significantly higher CIL scores than students with one or zero computers at home (Fraillon et al., 2020). Demographic and socio-economic factors are drivers of digital exclusion in terms of access (Sanz & Turlea, 2012). Research has also shown that young people with better access to ICT at home or at school, and those with a more positive attitude towards ICT, have greater digital skills (Haddon et al., 2020). Harris et al. (2017) studied the information technology (IT) usage of 1,351 Australian children aged between 6 and 17 years. In their research, they found socio-economic status to be a determinant of how children use IT. In high socio-economic neighbourhoods, children were involved in IT activities, reading, playing musical instruments and engaging in physical activities. By contrast, in low socio-economic neighbourhoods, children were more exposed to TV, electronic games, mobile phones and non-academic computer use at home. However, the authors did not address the digital divide in terms of ICT access, as they considered that the digital divide lay in how (not whether) children used devices.

Additionally, research by Livingstone et al. (2005) and Livingstone and Helsper (2007) examined inequalities in internet access and usage among children aged 9–19, using the UK Children Go Online survey. The results of this research showed that more deprived regions had lower levels of internet access. The same was true of children with disabilities, who also had lower levels of internet access. Ting-Feng et al. (2014) explored the digital divide among students with learning disabilities, and found that while there was no disadvantage in terms of internet access, there was in terms of digital literacy. Similar results were reported by Vicente and López (2010), who showed that people with disabilities are less confident about their online activities and skills. Jackson et al. (2008) and Judge et al. (2006) found a positive relationship between narrowing the digital divide, ICT use and academic performance. Finally, Chinn and Fairlie (2004) studied the determinants of computer and internet use in high-income and low-income countries, including a wide range of economic, demographic and policy factors. They found that the global digital divide was mainly explained by income disparities, communication infrastructures, access to electricity, the institutional environment and demographic characteristics (James, 2008). However, few studies have approached the digital divide from a cross-country perspective, as we do in this paper.

Importantly, none of the literature reviewed on digital exclusion uses up-to-date data to study the prevalence of digital deprivation across European countries and over time. Also, we have been unable to find any recent studies that tackle the socio-economic and demographic characteristics that define the phenomenon in Europe. Thus, we aim to fill this gap in the literature by providing a recent detailed account of who the digitally deprived children in Europe are and what socio-economic characteristics they share.

## Data

The data set used in this paper is the EU-SILC in its cross-sectional form, provided to researchers by Eurostat. This survey aims to collect comparable microdata on all aspects of Europeans’ living conditions, including (among other things) on income, material deprivation, labour market, demographic and educational characteristics, childcare and housing costs. The data is collected by national statistical bodies, following a common framework. However, a degree of flexibility is allowed: the information can be either extracted from registers or collected from interviews, using five possible modalities — face-to-face interview (PAPI or CAPI), telephone interview (CATI), self-administered by respondent or proxy interview. Generally, Eurostat gives priority to face-to-face personal interviews (GESIS, 2022). Most of the analysis focuses on data relative to 2019 as it is the last wave that allows a comparative analysis of most European countries.^4^ Data for Iceland and the UK is not provided, and so we use data relative to 2018 for these countries. The EU-SILC has several advantages for the purposes of our research: (i) it allows a comparative analysis across Europe, with evidence for 32 countries; (ii) it provides very detailed information on the socio-economic background of children, as it includes data on household income, parental characteristics (such as labour market attachment), household structure, material deprivation, etc.; and (iii) it allows us to track changes over time, as it covers a relatively long period — most countries have participated since 2004.

The information relating to digital deprivation is contained in two variables. *HS090* collects, at the household level, the answers to the question ‘Does your household have a computer?’ Household respondents can answer ‘yes’ or ‘no’. If the answer is negative, the question continues as follows: ‘If you do not have a computer: (a) Would you like to have it but cannot afford it or, (b) Do you not have one for other reasons, e.g. you do not want or need it?’^5^*PD080* collects, at the individual level, the answers to the question ‘Do you have an internet connection for personal use when needed?’ In this case, all adult members in the household can answer ‘yes’ or ‘no’. And, again, if the answer is negative, they are asked whether it is because of unaffordability or for some other reasons. The data documentation clarifies that such internet access can be via smartphone, other wireless handheld device (e.g. a tablet), video games console, laptop, desktop computer or TV.^6^ We define as ‘digitally deprived’ those children that either live in a household that cannot afford to have a computer and/or live with adults who cannot afford an internet connection.^7^

Importantly, there are other databases that collect a wider array of digital indicators; but in such cases we do not have as detailed information on the socio-economic background of children as the EU-SILC provides. Furthermore, this is the only data set that we know of that records enforced lack; thus, it is clearly stated that the members of the household would like to have a given item, but cannot afford it (Mack & Lansley, 1985; Marlier et al., 2007).^8^

Our sample considers children from above the age of 5 and below the age of 17, thus covering the period of compulsory education in the vast majority of countries analysed. We consider children who live with at least one parent.^9^ As Table 1 shows, the average age of the children was 11 years, and the parents were on average aged 42. Also, 48% of the sample were girls. Mean household size was 4.22 members, and 19% of children lived in a single-parent household.^10^ Furthermore, 20% of children in the sample were poor. Following the European Commission guidelines for the measurement of poverty in Europe, a household is defined as being in poverty if the equivalent household income is below 60% of the median of the same distribution. The modified OECD equivalence scale that gives a weight of 1 to the first adult, 0.5 to any other adults in the household and 0.3 to children below the age of 14 is used. Also, 6% of children live in ‘severe material deprivation’, according to the definition of the European Commission. That is, out of nine possible items, they lack at least four.^11^ Finally, 15.2% of the children have at least one parent of non-European immigrant origin; 13.1% cohabit with parents who had not acquired education above the level of primary school or compulsory lower secondary school (ISCED 2011, level 0–2); and 24.2% live in a large family, with at least three children under the age of 18 in the household.,^12^,^13^

**Table 1:** Summary statistics, school-aged children, Europe, 2019

Variable	Mean	Std. Dev.	Min	Max
Age	10.966	3.128	6	16
Parents’ average age	42.465	6.280	17	76
Female	0.480	0.500	0	1
Household size	4.217	1.191	2	22
Single-parent household	0.190	0.392	0	1
Poor	0.199	0.399	0	1
Materially deprived (severe)	0.060	0.238	0	1
Parents of non-European immigrant origin	0.152	0.359	0	1
Low-educated parents	0.131	0.337	0	1
Large family (3 + children)	0.242	0.428	0	1

Note: Data for the UK and Iceland refers to 2018

Source: Authors’ computation, using data from EU-SILC, 2019 (released November 2021)

## Digital Deprivation in Europe

This section presents the main results of our study. First, we discuss our findings regarding the prevalence of child digital deprivation across Europe and how it has changed over time. Then we present the results regarding the socio-economic characteristics that are associated with child digital deprivation both at the European level and by country cluster.

### Children’s Digital Deprivation across Countries and Over Time

**Fig. 1:**
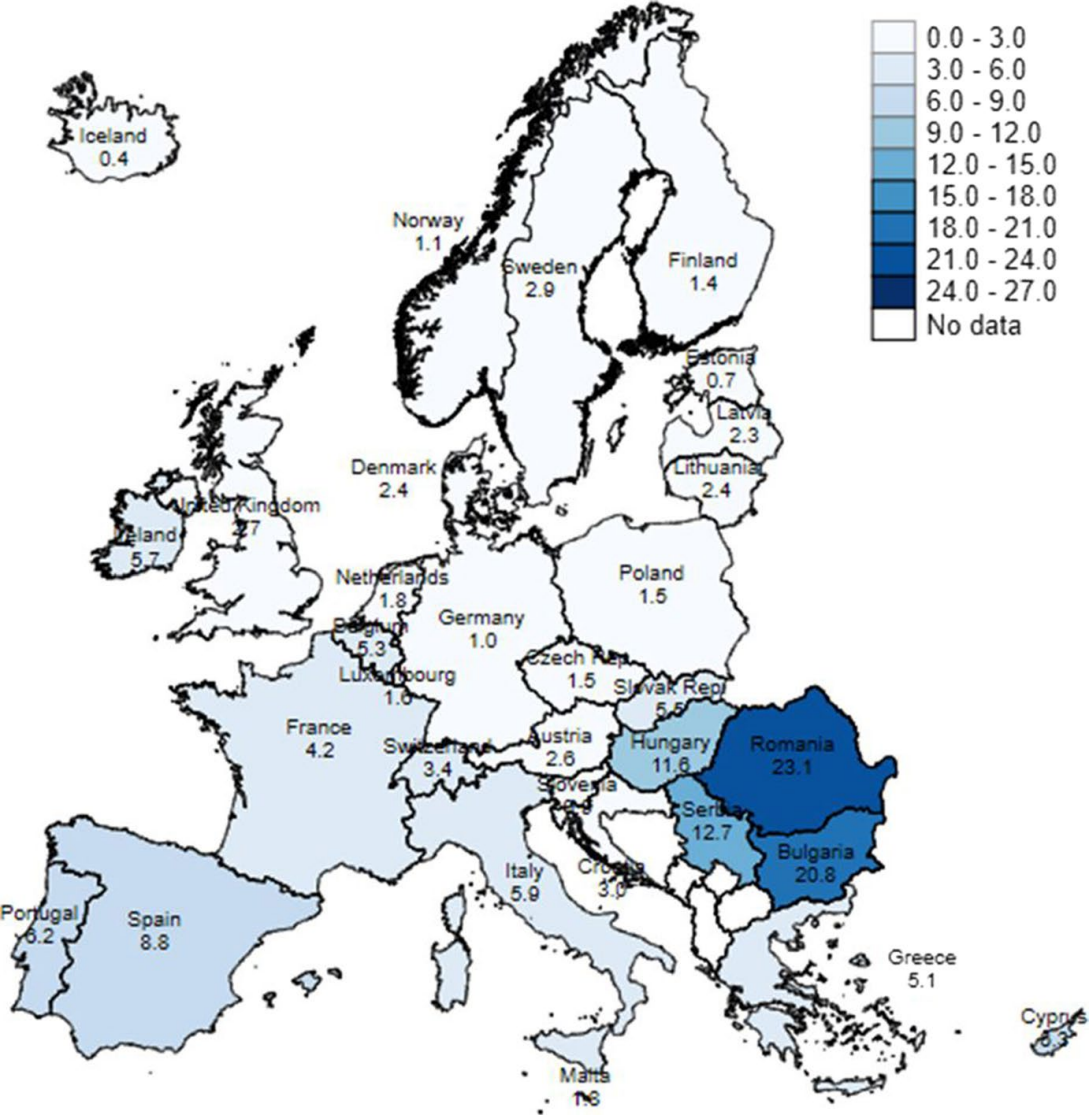
Percentage of digitally deprived school-aged children (6–16), Europe, 2019. Note: Data for the UK and Iceland refers to 2018. In Austria, Denmark, Estonia, Finland, Germany, Iceland, Luxembourg, Malta, the Netherlands, Norway and Slovenia, fewer than 30 observations define the digitally deprived population, and therefore the results should be interpreted with caution. Source: Authors’ computation, using data from EU-SILC, 2019 (released November 2021)

As mentioned above, 5.4% of school-aged children in Europe are digitally deprived, according to the latest data available — albeit the differences across countries are very large. Figure 1 shows the percentage of children who live in a household that cannot afford to have a computer and/or cohabit with adults who cannot afford to have an internet connection. The choropleth map shows two country clusters with a certain North–South divide. On the one hand, in Northern and Continental Europe, as well as in the Baltic countries and the UK, the percentages of digitally deprived children are very low — as low as 0.4% in Iceland, 0.7% in Estonia and 1.1% in Norway. None of the countries in this cluster have percentages above 3%. On the other hand, the prevalence of the phenomenon is much higher in the Mediterranean countries, and particularly in Eastern Europe (as indicated by darker colours on the map). In Romania, 23.1% of children are digitally deprived, and in Bulgaria 20.8%. The percentages are not as high in Hungary or Serbia, but more than 1 child in 10 is faced with the problem. Among the Mediterranean countries, it is in Spain that the percentage is highest (8.8%).^14^

**Fig. 2:**
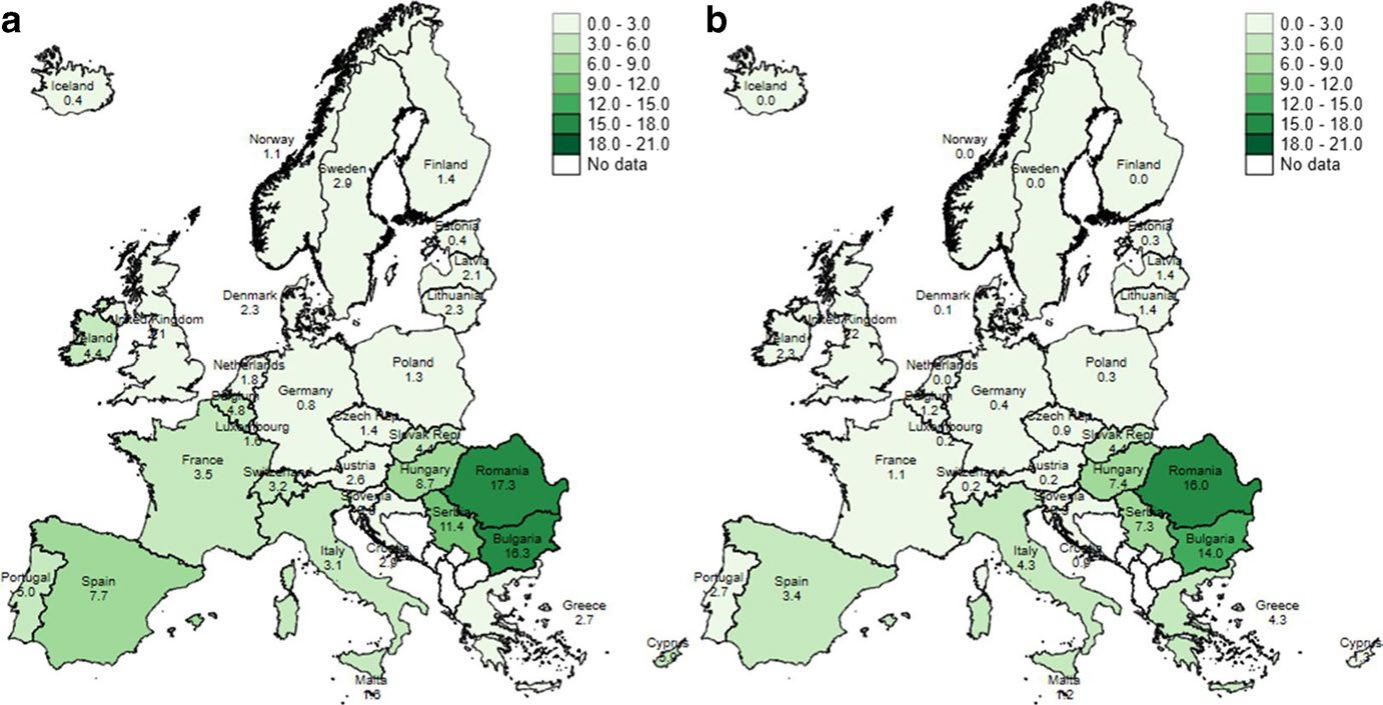
**a** Percentage of school-aged children (6–16) who live in a household that cannot afford a computer, Europe, 2019. **b** Percentage of school-aged children (6–16) who live in a household that cannot afford an internet connection, Europe, 2019. Note: The data for the UK and Iceland refers to 2018. In Austria, Denmark, Estonia, Finland, Germany, Iceland, Luxembourg, Malta, the Netherlands, Norway and Slovenia, fewer than 30 observations define the digitally deprived population; thus, the results should be interpreted with caution. Source: Authors’ computation, using data from EU-SILC, 2019 (released November 2021)

Figure 2a  and b show the relative importance of the two indicators used: Fig. 2a refers to computer unaffordability and Fig. 2b refers to internet connection unaffordability. The maps indicate that, of the two items, it is the inability to have a computer at home which mostly drives the overall results. In this case, it should be noted that again a certain North–South divide emerges, with a greater prevalence in Mediterranean and Eastern European countries. On the positive side, there are several countries where no household with children reports being unable to afford an internet connection at home — see, among others, Finland, Norway, Iceland and Austria.

**Fig. 3:**
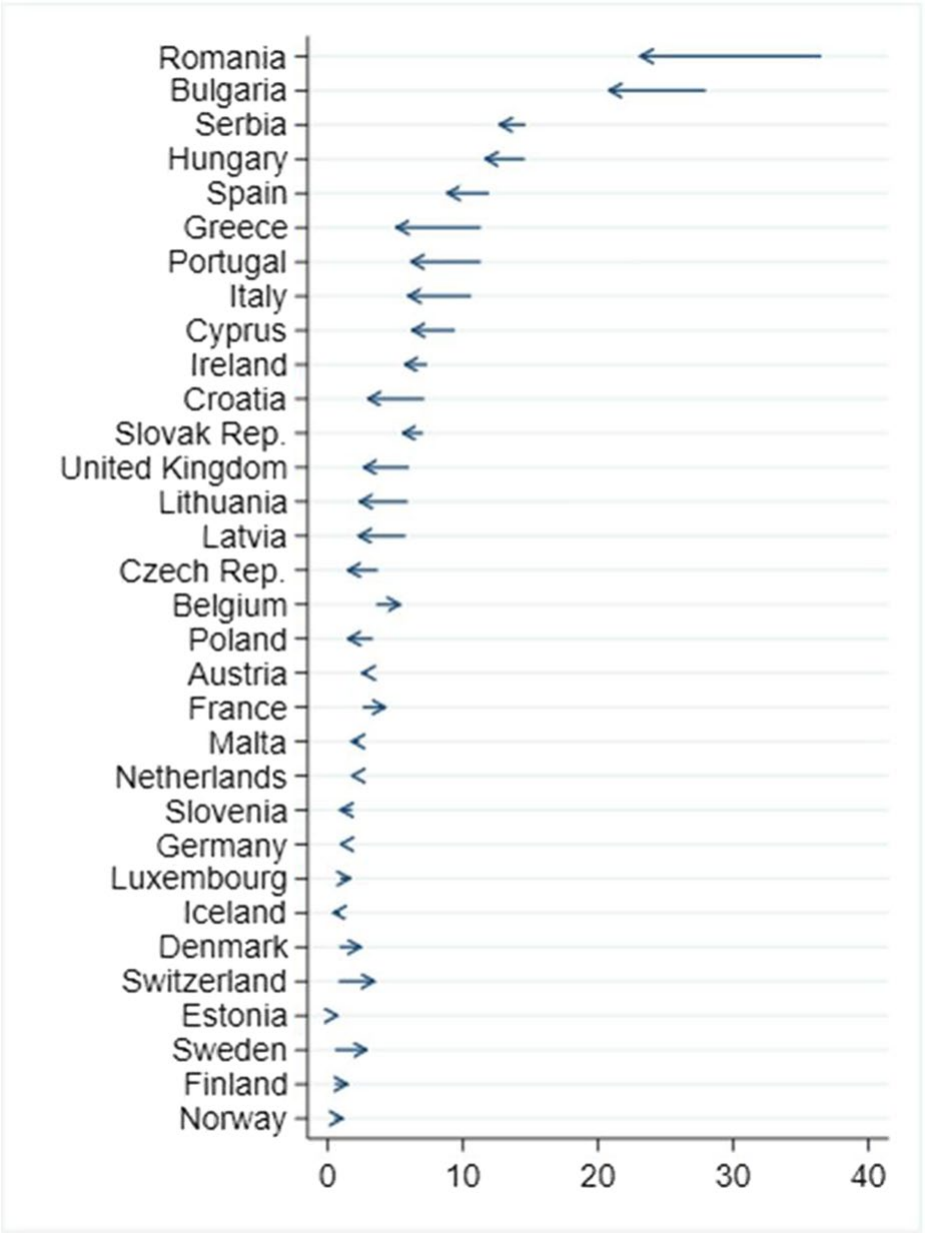
Percentage of school-aged children (6–16) digitally deprived, Europe, 2015–2019. Note: Data for the UK and Iceland refers to 2018 instead of 2019. In Austria, Denmark, Estonia, Finland, Germany, Iceland, Luxembourg, Malta, the Netherlands, Norway and Slovenia, fewer than 30 observations define the digitally deprived population in 2019 (or 2018); thus, the results should be interpreted with caution. Countries have been ranked according to the percentage of digitally deprived children in 2015. Source: Authors’ computation, using data from EU-SILC, 2015–2019

Despite the high figures for digital deprivation among school-aged children in Europe, an analysis of the trend over the past five years indicates that the great majority of countries — and particularly those most affected by the problem — have moved in the right direction. Figure 3 shows the percentage of children who were digitally deprived in 2015 and in 2019, with the arrow indicating the change in direction. For example, Romania has reduced the number of children affected by digital deprivation from 36.5% to 23.1% in this five-year period. In the case of Bulgaria, the change has been from 27.9% to 20.8%. Important advances have also taken place in Portugal, Greece and Italy, and to a smaller extent also in Serbia, Hungary and Spain. For the countries at the bottom of the figure, the change is not significant — largely because the problem was negligible in 2015.

### Who are the Digitally Deprived Children in Europe?

The previous section showed the great heterogeneity of digital deprivation prevalence among school-aged children in Europe, and how it has changed in the past five years. In this section, we aim to identify the socio-economic and demographic characteristics that define a digitally deprived child in Europe. Is it parental education? The number of children in the household? Or the fact that the family receives income below the poverty line? In our analysis, we consider six household characteristics that could potentially be associated with digital deprivation: the child lives (1) in a single-parent household; (2) in a poor family; (3) in a severely materially deprived household; (4) with at least one parent of non-European origin; (5) with parents that have at most lower secondary education; and (6) with at least two other siblings under the age of 18. In order to be able to work with a larger sample, and given that some characteristics relate to minorities, the results refer to the last five waves of data — that is, the period between 2015 and 2019.^15^ Furthermore, and on account of the small number of observations that define the digitally deprived population in some countries, as well as presenting the results at the European level, we show them by country cluster.^16^

**Fig. 4:**
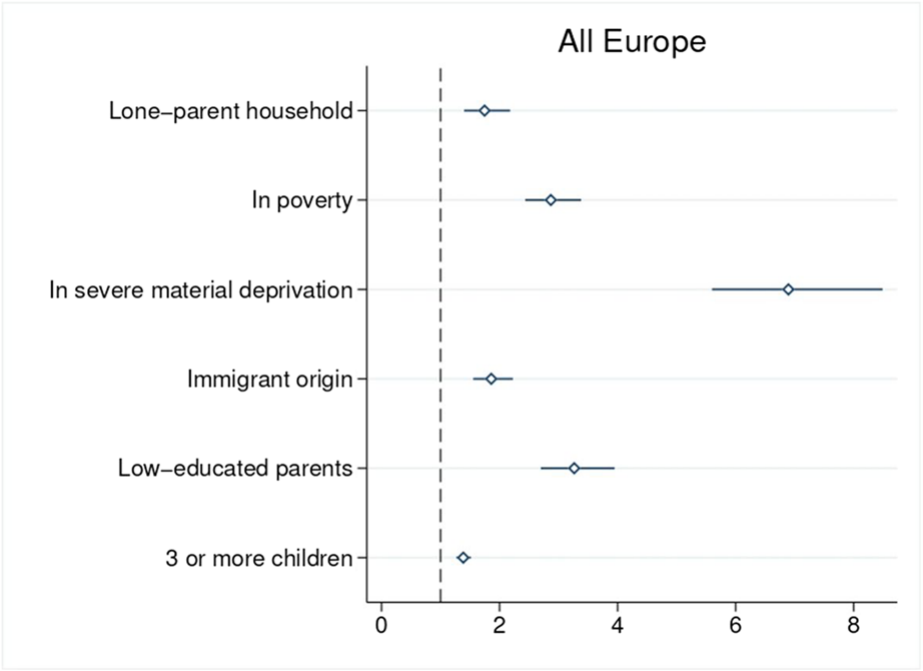
Logistic regressions (odds-ratios) for the probability of being digitally deprived, by socio-economic characteristics in school-aged children (6–16 years), Europe, 2015–2019. Note: Data for the UK and Iceland refers to the period 2015–2018. The horizontal line indicates confidence intervals at 95%. Source: Authors’ computation, using data from EU-SILC, 2015–2019

**Fig. 5:**
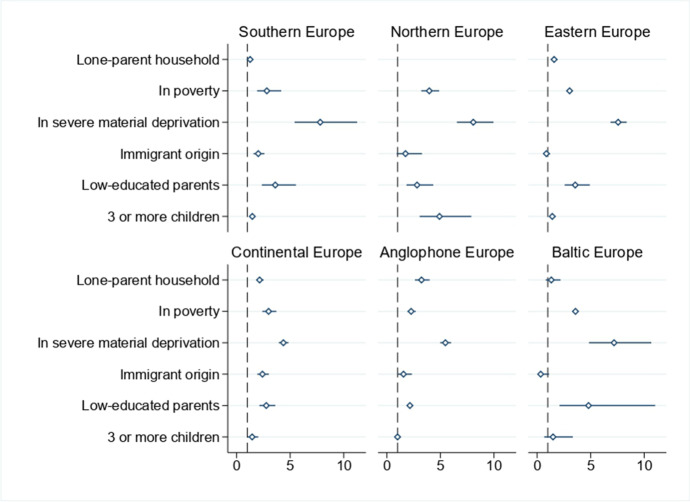
Logistic regressions (odds-ratios) for the probability of being digitally deprived, by socio-economic characteristics in school-aged children (6–16 years), European country clusters, 2015–2019. Note: Data for the UK and Ireland refers to the period 2015–2018. The horizontal line indicates confidence intervals at 95%. The result for lone-parent households in Northern Europe is not shown given that it has a very large confidence interval. Source: Authors’ computation, using data from EU-SILC, 2015–2019

Our systematic exploration of the demographic and socio-economic characteristics associated with digital deprivation is based on logistic regressions, which we present in Table 2 and in Figs. 4 and 5 in the form of odds ratios.^17^ Our dependent variable takes value 1 if the child is digitally deprived and 0 otherwise. Our parameters of interest are those associated with the six at-risk groups mentioned above. Control variables include individual characteristics of the child (gender, age and age squared), household size, parents’ average age (and its square), year dummies (to control for changes over time) and country dummies (to control for time-invariant country characteristics).^18^ Standard errors are robust and clustered at the country level.

**Table 2:** Logistic regression (odds-ratios) for the probability of being digitally deprived at the European level and by country cluster

Variables	All Europe	Northern Europe	Southern Europe	Eastern Europe	Continental Europe	Anglophone Europe	Baltic Europe
Age	1.0472	1.1021	1.0763***	0.9550	1.3019***	0.9660	1.4604
	(0.0581)	(0.1346)	(0.0240)	(0.0622)	(0.1007)	(0.0380)	(0.3686)
Age squared	0.9963	0.9936	0.9947***	1.0011	0.9857***	1.0009	0.9810*
	(0.0026)	(0.0055)	(0.0010)	(0.0028)	(0.0028)	(0.0017)	(0.0114)
Female	0.9694	1.1887	0.9842	1.0565*	0.8738*	0.8578***	0.9057**
	(0.0336)	(0.4188)	(0.0430)	(0.0323)	(0.0699)	(0.0010)	(0.0357)
Household size	1.0310	0.9197	0.9970	1.0981***	0.8823***	1.0599	0.9118
	(0.0260)	(0.1204)	(0.0145)	(0.0147)	(0.0397)	(0.0568)	(0.0951)
Parents’ average age	0.8031***	0.9953	0.8257***	0.8222***	0.7915***	0.7472***	0.9459
	(0.0309)	(0.0596)	(0.0605)	(0.0389)	(0.0168)	(0.0000)	(0.0699)
Parents’ average age squared	1.0023***	0.9994	1.0018**	1.0023***	1.0026***	1.0028***	1.0014***
	(0.0004)	(0.0005)	(0.0008)	(0.0005)	(0.0003)	(0.0000)	(0.0005)
Lone-parent household	1.7462***	3.8879	1.2614***	1.5904***	2.1469***	3.2343***	1.3308
	(0.1973)	(3.6571)	(0.1033)	(0.1439)	(0.1737)	(0.3481)	(0.3383)
In poverty	2.8687***	2.9111***	2.8227***	3.0366***	2.9861***	2.2683***	3.5868***
	(0.2404)	(0.7286)	(0.5601)	(0.1098)	(0.3309)	(0.2012)	(0.0852)
Severe material deprivation	6.8943***	7.3435***	7.8035***	7.5719***	4.3651***	5.4661***	7.1887***
	(0.7322)	(0.4638)	(1.4566)	(0.3838)	(0.2279)	(0.2587)	(1.4464)
Immigrant origin	1.8591***	1.9587***	2.0220***	0.8693	2.4139***	1.5559**	0.3411*
	(0.1704)	(0.4759)	(0.2564)	(0.1542)	(0.2704)	(0.3242)	(0.2068)
Low-educated parents	3.2647***	2.3170***	3.6107***	3.5632***	2.7698***	2.1542***	4.8051***
	(0.3170)	(0.6851)	(0.7879)	(0.5891)	(0.3746)	(0.0608)	(2.0360)
3 or more children	1.3854***	3.5050***	1.4673***	1.4230***	1.4534**	1.0018	1.4919
	(0.0631)	(0.5476)	(0.0699)	(0.1403)	(0.2437)	(0.1232)	(0.6122)
Observations	333,453	40,743	92,901	92,969	67,365	18,815	20,660
Year FE	Yes	Yes	Yes	Yes	Yes	Yes	Yes
Country FE	Yes	Yes	Yes	Yes	Yes	Yes	Yes

Note: Standard errors are in parenthesis. ***, ** and * represent statistical significance at 1%, 5% and 10%, respectively. Data for the UK and Iceland refers to the period 2015–2018

Source: Authors’ computation, using data from EU-SILC, 2015–2019

As shown in the first column of Table 2 and in Fig. 4, at the European level one characteristic clearly stands out as being very closely linked to children’s digital deprivation: living in severe material deprivation. On average across Europe, that increases the risk of suffering digital deprivation by a factor of 6.9 among school-aged children. Being poor and having low-educated parents are also relevant factors — these variables multiply the risk of being digitally deprived by a factor of 2.9 and 3.3, respectively. All other risk factors considered are positive (albeit at a lower level) and statistically significant at 99%. As for the control variables, we find no statistically significant differences between boys and girls, while the risk of being digitally deprived increases with age and decreases with parental age.

Next, we move to the results by country cluster (see Fig. 5). With very few exceptions (remarked on below), we find that the large bulk of the risk factors considered are positively linked to digital deprivation — though the strength of the association varies by context. In all groups of countries, the characteristic most strongly associated with digital deprivation is living in a household with severe material deprivation. For example, in Eastern Europe it multiplies the probability of being digitally deprived by 7.6; in the Baltic countries by 7.2 and in Continental Europe by 4.4. Cohabiting with low-educated parents is of particular importance in Eastern Europe, the Mediterranean countries and the Baltic area: in all those clusters, the probability of being digitally deprived increases by at least a factor of 3.5. Poverty is also a strong determinant of digital deprivation among school-aged children, though with a similar effect in all the country clusters analysed. With the sole exception of Northern Europe (which shows high risk), living in a large family has a more muted effect, and does not differ statistically from zero in the English-speaking countries. The same is true for living in a single-parent household, with relatively low risk in Southern and Eastern Europe and in the Baltic countries. In this last case, the associated odds ratio is not precisely estimated. Finally, and interestingly, having parents of non-European immigrant origin reduces the likelihood of digital deprivation in Eastern Europe and the Baltic area, while it increases the probability in all other contexts.

For the interested reader, Table 5 in the Appendix presents qualitative results from similar regressions as those in Table 2, but at country level, with the objective of providing a more nuanced picture of the determinants of digital deprivation across Europe.^19^ In this case, we only consider countries where the digitally deprived sample of children is above 150 observations for the five-year period. The main takeaway from these results is that severe material deprivation, poverty and low parental education are positively associated with digital deprivation in all the countries analysed, with the results statistically significant at 99% confidence level in all cases. The degree of association with digital deprivation for the rest of the characteristics varies much more, with the results less precisely estimated.

## Conclusions

This paper provides a detailed account of the digitally deprived children in Europe. We use the cross-sectional form of the latest wave available of the European Union – Statistics on Income and Living Conditions (EU-SILC), which refers to 2019. To the best of our knowledge, this is the only database that records enforced lack, and it allows us to identify households that are digitally deprived because of unaffordability. We consider lack of access to a computer and lack of access to an internet connection at home.

We find that 1 child in 20 in Europe is digitally deprived, with substantial differences from country to country. For example, in Romania, 3 children in 10 live in digital deprivation. In Bulgaria, the figure is 2 in 10. Thus, we document an important problem of access to the tools necessary for education in today’s Europe: for many children, having a computer connected to the internet makes all the difference between keeping up with their education and falling badly behind. As a result, inequalities are likely to be exacerbated.

Of the two items considered (access to a computer and internet access), inability to afford a computer is far more prevalent than inability to afford an internet connection. The phenomenon is particularly widespread in Southern and Eastern European countries, and it particularly affects children who live with low-educated parents, in a poor household and/or in severe material deprivation. Nonetheless, the heterogeneity of the characteristics that describe a digitally deprived child across countries is worth noting. For example, in Eastern Europe, having parents of non-European immigrant origin is not associated with a higher probability of being a digitally deprived child, whereas in the remaining country clusters it is.

Computer and internet access can benefit children. Those who have access can be said to have a better opportunity to develop their interests, confidence and skills; as a result, these children and young people can benefit more fully from digital technologies, because they have both a better understanding of them and the opportunity to develop the digital competences required in today’s world. Digital exclusion can impose a burden on children in terms of inclusion and participation in the online environment. Access to ICT can also be important in terms of mental health, which has been found to be worse among those children who experience problems of digital exclusion (Metherell et al., 2021).

Our study highlights the fact that access to a computer and to an internet connection are not guaranteed for all European children. It provides new findings on who these children are and where they live. In this way, we aim to contribute to the creation of evidence-based policies that can play a part in reducing the existing digital inequalities in access. Current and future policy efforts should target and support children who share the socio-economic characteristics associated with digital deprivation. If we want to achieve equality of opportunity in education, we should begin by providing equal access to education, which now implies having access to a computer and internet connection. Furthermore, schools, as a part of communities, carry an element of continuity; both in time of crisis and in the future, there exists the challenge of how to secure educational activities to support learning and continuity in children’s lives. Therefore, it is crucial for children to have access not only to the internet, but also to the digital tools that are essential for their education and that can further the development of their digital skills. Digital deprivation limits not only children’s access to information, but also their opportunity to develop the digital skills they need in the 21st century — technical, information, communication, collaborative, creative, critical-thinking and problem-solving (van Laar et al., 2017). Furthermore, van Laar et al. (2017) remind us that ‘the dynamic changes in the types of jobs demanded by the knowledge society pose serious challenges to educational systems, as they are currently asked to prepare young people for jobs that may not yet exist’ (p. 584). Certainly, ensuring that children have access to digital technology in order to start developing these crucial digital skills is an important first step.

Finally, our study is limited by the information included in the EU-SILC database regarding new technologies. To improve the monitoring of the situation of children, both during and after the COVID-19 pandemic, the database should incorporate more variables, including (but not limited to) whether the household has more than one computer available; whether children own or can use any other devices; internet speed; and the cost of internet access. It is of the utmost importance to learn the extent to which devices in the household are truly available to children; how frequently those devices are used and for how long; the rules governing sharing of the devices; and even children’s own satisfaction with the use of technology. Certainly, the increasing prominence of digital technology, which is even more evident following the COVID-19 pandemic, means that it will be ever more important to identify the extent and depth of digital deprivation across Europe. This knowledge will allow governments to target areas of need and to develop appropriate legislation to combat deepening societal inequalities.

## Figures and Tables

**Fig. 6:**
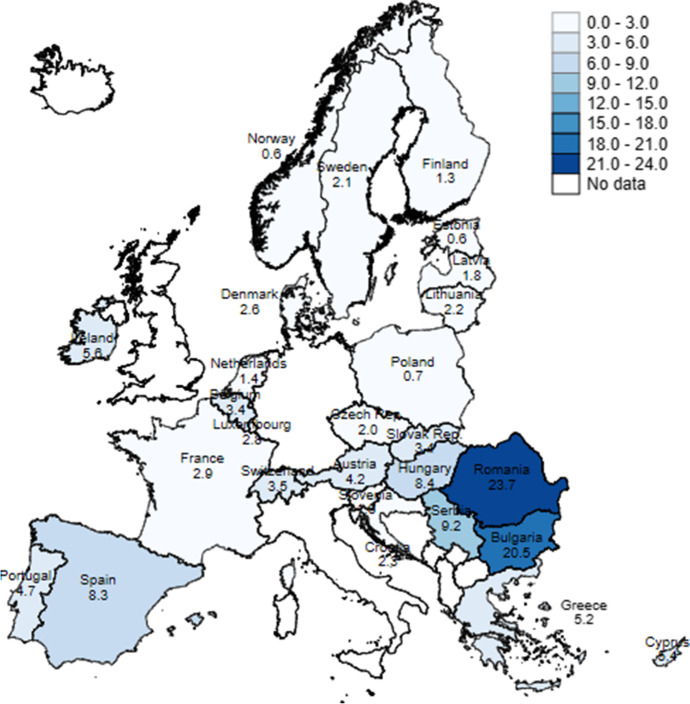
Percentage of digitally deprived school-aged children (6–16), Europe, 2020. Note: Data not available for Germany, Iceland, Italy, Malta and the UK. In Denmark, Estonia, Finland, Latvia, Lithuania, Luxembourg, the Netherlands and Norway, fewer than 30 observations define the digitally deprived population; thus, the results should be interpreted with caution. Source: Authors’ computation, using data from EU-SILC, 2020 (released April 2022)

## Data Availability

The data that supports the findings of this study (EU-SILC) is available from Eurostat, but restrictions govern the availability of the data, which was used under licence for the current study and is not publicly available.

## Appendix

**Table 3:** EU-SILC availability table, Europe, 2015–2020

	2015	2016	2017	2018	2019	2020
Austria	✓	✓	✓	✓	✓	✓
Belgium	✓	✓	✓	✓	✓	✓
Bulgaria	✓	✓	✓	✓	✓	✓
Croatia	✓	✓	✓	✓	✓	✓
Cyprus	✓	✓	✓	✓	✓	✓
Czech Rep	✓	✓	✓	✓	✓	✓
Denmark	✓	✓	✓	✓	✓	✓
Estonia	✓	✓	✓	✓	✓	✓
Finland	✓	✓	✓	✓	✓	✓
France	✓	✓	✓	✓	✓	✓
Germany	✓	✓	✓	✓	✓	
Greece	✓	✓	✓	✓	✓	✓
Hungary	✓	✓	✓	✓	✓	✓
Iceland	✓	✓	✓	✓		
Ireland	✓	✓	✓	✓	✓	✓
Italy	✓	✓	✓	✓	✓	
Latvia	✓	✓	✓	✓	✓	✓
Lithuania	✓	✓	✓	✓	✓	✓
Luxembourg	✓	✓	✓	✓	✓	✓
Malta	✓	✓	✓	✓	✓	
Netherlands	✓	✓	✓	✓	✓	✓
Norway	✓	✓	✓	✓	✓	✓
Poland	✓	✓	✓	✓	✓	✓
Portugal	✓	✓	✓	✓	✓	✓
Romania	✓	✓	✓	✓	✓	✓
Serbia	✓	✓	✓	✓	✓	✓
Slovak Rep	✓	✓	✓	✓	✓	✓
Slovenia	✓	✓	✓	✓	✓	✓
Spain	✓	✓	✓	✓	✓	✓
Sweden	✓	✓	✓	✓	✓	✓
Switzerland	✓	✓	✓	✓	✓	✓
United Kingdom	✓	✓	✓	✓		

Source: Authors’ computation, using data from EU-SILC, 2015–2020.

**Table 4:** Summary statistics, school-aged children, Europe, 2015–2019

Variable	Mean	Std. Dev	Min	Max
Age	10.948	3.159	6	16
Parents’ average age	42.174	6.250	17	80
Female	0.480	0.500	0	1
Household size	4.249	1.215	2	22
Single-parent household	0.187	0.390	0	1
Poor	0.210	0.407	0	1
Materially deprived (severe)	0.081	0.273	0	1
Parents of non-European immigrant origin	0.142	0.349	0	1
Low-educated parents	0.143	0.351	0	1
Large family (3 + children)	0.244	0.430	0	1

Note: Data for the UK and Iceland refers to 2015–2018 instead of 2015–2019

Source: Authors’ computation, using data from EU-SILC, 2015–2019

**Table 5:** Results of the logistic regressions (odds ratios) for the probability of being digitally deprived at country level, Europe, 2015–2019

	Lone-parent household	In poverty	In severe material deprivation	Immigrant origin	Low-educated parents	3 or more children
Austria		+ ***	+ ***	+ ***	+ ***	
Belgium	+ ***	+ ***	+ ***	+ ***	+ ***	
Bulgaria		+ ***	+ ***		+ ***	+ ***
Croatia		+ ***	+ ***		+ ***	
Cyprus	+ ***	+ ***	+ ***	+ ***	+ ***	
Czech Rep	+ ***	+ ***	+ ***		+ **	
France	+ ***	+ ***	+ ***	+ ***	+ ***	+ ***
Greece	+ ***	+ ***	+ ***	+ ***	+ ***	
Hungary	+ ***	+ ***	+ ***		+ ***	+ ***
Ireland	+ ***	+ ***	+ ***	- ***	+ ***	- **
Italy		+ ***	+ ***	+ ***	+ ***	+ ***
Latvia	+ **	+ ***	+ ***		+ ***	
Lithuania		+ ***	+ ***	- ***	+ ***	+ ***
Poland	+ ***	+ ***	+ ***		+ ***	
Portugal	+ ***	+ ***	+ ***	- ***	+ ***	+ ***
Romania	+ ***	+ ***	+ ***		+ ***	+ ***
Serbia	+ ***	+ ***	+ ***	- **	+ ***	
Slovenia	+ ***	+ ***	+ ***	+ ***	+ ***	
Slovak Rep	+ ***	+ ***	+ ***		+ ***	+ **
Spain	+ **	+ ***	+ ***	+ ***	+ ***	+ ***
UK	+ ***	+ ***	+ ***	+ ***	+ ***	

Note: ***, ** and * represent statistical significance at 1%, 5% and 10%, respectively. Data for the UK and Iceland refers to the period 2015 to 2018. We do not consider countries with fewer than 150 observations for the sample of children in digital deprivation

Source: Authors’ computation, using data from EU-SILC, 2015–2019

## Footnotes

1 UNESCO (2020) estimates that school closures in March 2020 affected 290.5 million students across the world.

2 As we describe in the next section, the database used in the analysis is the European Union – Statistics on Income and Living Conditions (EU-SILC), so we calculate the digital divide according to the information available.

3 Maiti and Awasthi (2020) also find a positive relationship between ICT exposure and an aggregate indicator of well-being and progress in 67 countries.

4 At the time of writing, the latest release of data (provided to researchers in April 2022) includes information for 2020, but data is not available for a number of countries (Germany, Iceland, Italy, Malta and the United Kingdom) — see Table 3 in the Appendix; therefore, we have opted to use data from 2019 in the main text and keep the results for 2020 in the Appendix. Importantly, it should be noted that our results barely change when we use data for 2020 for those countries for which information is available.

5 According to the data documentation, ‘possessing the item does not necessarily imply ownership: the item may be rented, leased, provided on loan or shared with other households in (e.g.) a complex apartment and not necessarily owned. If the item is shared between households, the answer is YES if there is adequate/easy access (i.e. household can use the durable whenever it wants) and NO otherwise … A computer includes a portable computer or a desktop computer, but does not include machines dedicated to video games that do not have any broader functionality. If a computer is provided ONLY for work purposes, this does not count as possessing the item’ (Eurostat, 2020, p. 196).

6 In the data documentation, more detail is provided: ‘Example of internet activities for personal use: social networking, sending/receiving emails, using services related to travel and accommodation, creating web pages, blogs, internet banking, reading or downloading online music, video, news, etc. looking for information, telephoning or making video calls, buying/selling goods or services, taking part in online consultations or voting on civic or political issues, etc. The household member is considered to have internet connection for personal use at home only if all the needs for personal use he/she are fully fulfilled by this connection.’(Eurostat, 2020, p. 345).

7 Guio et al. (2012) considered that a household was deprived only if it lacked both a computer and an internet connection. Guio et al. (2017) only accounted for the lack of an internet connection under the argument that many individuals can now access the internet using other devices such as smartphones or tablets. While this is true, in this study, we want to consider both indicators given the large number of European children that now receive part or all their teaching online.

8 Such information allows us to disregard families that, because of their life style, do not want to have an internet connection and/or a computer.

9 Only 0.95% of children in our sample live in a household where neither parent is present.

10 The great majority of children in single-parent households live with the mother (83.16%).

11 The items that the European Commission considers to measure severe material deprivation include the impossibility to: (i) avoid arrears in rent, mortgage or utility bills; (ii) keep the home adequately heated; (iii) face unexpected expenses; (iv) eat meat or proteins regularly; (v) go on holiday; (vi) have a television set; (vii) have a washing machine; (viii) have a car; and (ix) have a telephone.

12 In single-parent households, we only consider the education of the mother or the father cohabiting with the child.

13 We do not distinguish between urban and rural areas, because the information is missing for 10.01% of the sample.

14 Figure 6 in the Appendix shows the percentage of digitally deprived children in Europe in 2020 for those countries with available data. The change between 2019 and 2020 is not statistically significant in most countries, except for Belgium, Portugal, Spain and Sweden (where we can document a slight decrease in the percentage of digitally deprived children) and Slovenia (where a small increase is to be found).

15 Table 4 in the Appendix presents summary statistics for this five-year sample.

16 We consider six country clusters: Southern Europe (Spain, Greece, Italy, Portugal, Cyprus and Malta), Northern Europe (Finland, Sweden, Norway, Iceland and Denmark), Eastern Europe (Hungary, Poland, the Czech Republic, Romania, Serbia, Croatia, Slovenia, Slovakia and Bulgaria), Continental Europe (Belgium, Austria, Switzerland, Germany, France, Luxembourg and the Netherlands), the Anglophone countries (the United Kingdom and Ireland) and the Baltic area (Estonia, Lithuania and Latvia).

17 An odds ratio above the value of 1 implies that a given characteristic is positively associated with digital deprivation, while a value below 1 implies a negative association.

18 In single-parent households, average age refers to the age of the parent present in the household.

19 Detailed results at country level are available from the authors on request.

## Code Availability

The Stata® code used is available from the authors upon request.
